# Green Sorbitol- and Isosorbide-Based Flame Retardants for Cotton Fabrics

**DOI:** 10.3390/ma14216375

**Published:** 2021-10-25

**Authors:** David De Smet, Madeleine Wéry, Miriam Bader, Ines Stachel, Michael Meyer, Myriam Vanneste

**Affiliations:** 1 Centexbel, Technologiepark 70, 9052 Zwijnaarde, Belgium; mw@centexbel.be (M.W.); mv@centexbel.be (M.V.); 2 FILK Freiberg Institute gGmbH, Meißner Ring 1-5, 09599 Freiberg, Germany; miriam.bader@filkfreiberg.de (M.B.); ines.stachel@filkfreiberg.de (I.S.); michael.meyer@filkfreiberg.de (M.M.)

**Keywords:** flame retardant, biobased, cotton, textile, sorbitol, isosorbide

## Abstract

Flame retardancy is often required in various textile applications. Halogenated flame retardants (FR) are commonly used since they have good FR performance. Several of these components are listed under REACH. Halogen-free FR compounds have been developed as alternatives. So far, not many biobased FR have made it to the market and are being applied in the textile sector, leaving great opportunities since biobased products are experiencing a renaissance. In this study, renewable FR based on sorbitol and isosorbide were synthesised. The reaction was performed in the melt. The resulting biobased FR were characterised via FT-IR, thermogravimetric analysis (TGA) and X-ray fluorescence (XRF). Cotton fabrics functionalized with the developed biobased FR passed ISO 15025 FR test. After washing, the FR properties of the fabrics decreased (longer afterflame and afterglow time) but still complied with ISO 15025, indicating the biobased FR were semi-permanent. The amount of residue of modified sorbitol and isosorbide measured at 600 °C in air was 31% and 27%, respectively. Cotton treated with biobased modified FR showed no ignition during cone calorimetry experiments, indicating a flame retardancy. Furthermore, a charring of the FR containing samples was observed by means of cone calorimetry and TGA measurements.

## 1. Introduction

Textiles are often finished with water/oil repellent, antimicrobials and FR products. Alternative processes and products are explored to lower the environmental impact of textile finishing. Much current research focuses on green and environmentally friendly antimicrobial products [1,2]. Relating to the upcoming REACH regulation, alternative repellent products are also examined to replace fluorocarbons, which are proven to be persistent, bioaccumulative and toxic, by looking to different chemistry or study the structure of fabrics [3,4].

The synthesis and application of FR in various materials raised the awareness of public bodies to limit the use or prohibit the use of some FR due to their (environmental) toxicity. Especially some halogenated FR are persistent, bioaccumulative and release toxic and corrosive gases in case of fire [5,6]. Legal regulations and the green awareness of consumers are major drivers to investigate alternative, environmentally friendly and non-toxic FR. Given its importance to consumer safety, fire retardant textiles are one of the fastest growing sectors in industrial textiles. Three approaches can be considered to reduce the flammability of textiles: (i) to use inherently flame retardant textiles (e.g., Trevira and Nomex); (ii) to chemically modify existing textiles; (iii) to incorporate FR in synthetic fibres and/or to make specific surface treatments [7]. Currently used FR include mostly halogenated (bromine or chlorine), inorganic salts and nitrogen/phosphorus based substances. Halogenated FR are frequently used in textile industry due to their very good FR properties. Often antimony trioxide is added to introduce synergistic FR effects. However, antimony trioxide is suspected to cause cancer and several halogenated FR (e.g., decabromodiphenyl oxide) are subject of regulation due to their persistent and bioaccumulative properties [8,9]. Therefore, polymeric halogen FR have been developed. However, the environmental fate or adequate processing of halogens at the end of life is still challenging.

Substances based on nitrogen and phosphorus are explored as alternative halogen-free FR. Both nitrogen- and phosphor-containing molecules are used together since they have synergistic flame retardant effects. Nitrogen compounds will show a blowing effect and the phosphorus compounds will form a char protecting the underlying material from the flame. Often used nitrogen-/phosphorus-based FR include ammonium (poly)phosphate, guanidine or ethylenediamine phosphate and melamine as nitrogen component.

To date, only few biobased FR are in use in the textile sector, leaving great opportunities. That is why polymers and additives made from renewable raw materials are current topics of many research projects, including biobased FR. Organic compounds containing nitrogen and/or phosphorus are possible candidates to introduce flame retardant properties to textiles. A distinction can be made between biobased materials examined as FR without any chemical modification (e.g., deoxyribonucleic acid (DNA), proteins and phytic acid) and biobased materials chemically modified with phosphorus- and/or nitrogen-containing groups. Vahabi et al. wrote a comprehensive overview of biobased FR in different applications [10]. Phytic acid is one of the widely examined FR to treat textiles owing to its high phosphorus content, abundance, non-toxicity and biocompatibility [11]. However, due to the acidity of phytic acid, fabrics treated with this biobased potent FR showed a decrease in mechanical properties (degradation of cellulose). This can be avoided by using ammonium phytate. Ammonium phytate was grafted on cellulose. The resultant grafted cotton fabrics exhibited superior flame retardancy displaying a limiting oxygen index (LOI) value of 43% (compared to 18% for untreated cotton) and a decrease of char length of 90% compared to untreated cotton fabric [12]. Tang et al. described the use of phytic acid as flame retardant for polylactic acid (PLA) nonwoven. LOI values increased with increasing content of phytic acid. None of the treated samples showed afterflame time [13]. The flame retardant properties of a mixture of phytic acid and triethanolamine was also assessed on cotton fabrics. It was found that finished samples had a significant higher LOI (32%) than neat cotton fabric (18%) [14]. Alongi et al. reported that DNA can be applied as green FR for cotton fabrics. LOI values significantly increased, burning rate slowed down and no flame ignition was observed. Additionally, the flammability of DNA-treated cotton fabrics was evaluated. Above a 10 wt% add-on, the DNA coating extinguished cotton as soon as the flame was removed [15]. Similarly, whey protein coatings enabled to slow down the burning rate and hence to increase the burning time [16].

Biobased polyols are studied as FR precursors. The molecules are chemically modified, e.g., phosphorylated, to enhance the flame retardant effect. Lignins as well as chemical modifications of lignins are extensively studied as FR since lignin is one of the most abundant natural polymers [17,18,19]. Results showed that the incorporation of untreated lignins led to a flame retardant action in PLA caused by the formation of char but also by a significant loss of the thermal stability of PLA. Furthermore, both phosphorus and nitrogen chemically treated lignins, were found to limit PLA thermal degradation during melt processing and to significantly improve flame retardant properties allowing to reach V0 classification at UL-94 [20]. However, often toxic chlorinated solvents and/or toxic phosphoryl chloride are used in the phosphorous modification of lignin. Besides, a biobased flame retardant based on vanillin was synthesised and assessed in PLA. With only 5 wt% loading of the phosphorus modified vanillin, PLA passed UL-94 V-0 classification [21].

Other sources of biobased materials include polysaccharides and vegetable oils. Vegetable oils are often used for biobased polyurethane synthesis. The application of a novel ricinoleic-acid-based phosphorus and nitrogen-containing FR polyol during synthesis of polyurethane sealant was reported. The ricinoleic acid based polyol enhanced the thermal stability and promoted char formation of the resulting polyurethane sealant [22]. Diphenolic acid is a biobased diol obtained from the reaction between levelunic acid and phenol. A polyphosphonate based on diphenolic acid was described and applied as FR. Blends of PLA with phosphorus modified diphenolic acid (2, 4 and 6%) were produced and the LOI was measured. PLA containing the polyphosphonate showed significant higher LOI. The fire behaviour was assessed via the vertical UL 94 test. PLA with levels equal or above 4% of the synthesized polyphosphonate were classified as V0, while neat PLA failed (no rating could be assigned) [23]. Furthermore, the use of FR based on isosorbide were reported. Isosorbide was converted to a variety of bis-phosphorous esters via either direct phosphorylation (using phosphoryl chloride) or the Atherton–Todd procedure [24]. Boday et al. prepared a polyphosphonate via a condensation polymerization reaction between isosorbide and phenylphosphonic dichloride in the presence of N,N-dimethylaminopyridine. Afterwards, the polyphosphonate was blended with PLA. UL-94 vertical flame tests were carried out to assess the flame-retardant properties. Neat PLA burned completely and could not be rated. Blends of PLA with either 5, 10, or 15% of polyphosphonate exhibited low extinguishing time. The composition with 15% of polyphosphonate obtained V0-rating, while for the other compositions V2-rating was achieved. However, increase of polyphosphonate content had negative effect on mechanical properties [25]. Isosorbide is a difunctional alcohol prepared from D-sorbitol, which is obtained by catalytic hydrogenation of D-glucose, which is in turn produced by hydrolysis of starch. The synthesis, using water as solvent, and application (pad-dry-cure) of a sorbitol-based flame retardant on lyocell fibres was reported. Vertical combustion and limited oxygen index results indicate that the treated lyocell fibres exhibited good flame retardant properties [26].

This paper aims at studying the solvent-free synthesis of nitrogen and phosphorus modified sorbitol and isosorbide using nontoxic raw materials. Sorbitol and isosorbide were modified in a two-step method and applied on cotton in a dyeing bath. A silicone-free defoamer was added to reduce foaming during synthesis. Dicyandiamide was used as the catalyst to promote linking of the flame retardant to the cotton fibres. The flame-retardant properties of the cotton treated with modified sorbitol were examined.

## 2. Materials and Methods

### 2.1. Materials

Sorbitol, isosorbide, phosphoric acid (85%), dicyandiamide and urea were purchased from Sigma-Aldrich (Darmstadt, Germany). Respumit NF01 was sampled by Tanatex Chemicals (Ede, The Netherlands). Woven cotton fabric (150 g/m^2^), used for garments and technical applications, was purchased from Utexbel (Ronse, Belgium).

### 2.2. Synthesis of Isosorbide Based Flame Retardant

The synthesis of isosorbide was based on a protocol published by Wan et al. for the synthesis of a flame retardant based on D-panthenol, with some minor modifications [27]. 109.63 g (0.75 mol) of isosorbide and 172.90 g (1.5 mol) phosphoric acid were mixed in a beaker with mechanical stirring. Then, the temperature was raised to 110 °C and held for 2.5 h. A viscous white liquid intermediate was obtained. Subsequently, 180.2 g (3 mol) ureum was added. The mixture was heated up to 120 °C. The formulation tends to foam and therefore 0.3% of Respumit NF01 defoamer was added. A viscous liquid was obtained. The product was dried in a vacuum oven at 70 °C. Yield: 96%

### 2.3. Synthesis of Sorbitol-Based Flame Retardant

A mixture of 45.54 g (0.25 mol) of sorbitol and 172.90 g (1.5 mol) phosphoric acid was made in a beaker with magnetic stirring. Then, the temperature was raised to 110 °C and held for 2.5 h. After the esterification reaction, 180.2 g (3 mol) urea was added. The mixture was heated up to 130 °C. The formulation tends to foam and therefore 0.3% of Respumit NF01 defoamer was added. A white solid product was obtained which was ground. The product was dried in a vacuum oven at 70 °C. Yield: 98%.

### 2.4. Finishing Cotton Fabrics

The sorbitol and isosorbide based FR were dissolved in water at a level of 40%. Ten percent dicyandiamide was added to the solution. Dicyandiamide was used as the catalyst to promote linking of the flame retardant to the cotton fibres. Dicyandiamide does not dissolve completely at room temperature, but dissolved while heating to 70 °C. Cotton samples with dimensions of 25 by 20 cm were immersed in dyeing baths with 100 mL solutions of flame retardant and dicyandiamide during 2 h at 70 °C in an IR dyer. Afterwards, the cotton fabrics were dried 1 min at 110 °C and cured for 4 min at 170 °C.

### 2.5. Characterisation

Fourier transform infrared spectra (FTIR in µ-ATR mode) were recorded using a Nicolet 6700 spectrometer from Thermo Fisher Scientific (Waltham, MA, USA). A spectral range from 500 cm^−1^ to 4000 cm^−1^ with a resolution of 4 cm^−1^ was applied. The infrared analysis was used to characterise the modified sorbitol and isosorbide as well as cotton fabrics treated with modified sorbitol and isosorbide.

X-ray fluorescence (XRF) measurements were conducted to determine the amount of phosphorus in sorbitol and isosorbide prior and after modification. The measurements were performed in helium atmosphere on an ARL Quant’x Energy Dispersive X-ray Fluorescence (EDXRF) system from Thermo Fisher Scientific (Waltham, MA, USA) using the Thermo Scientific UniQuant software (version 5) from Thermo Fisher Scientific (Waltham, MA, USA). Likewise, cotton fabrics treated with modified sorbitol and isosorbide were analysed by means of XRF.

Using a Q500 thermogravimetric analyser from TA Instruments (Asse, Belgium), thermogravimetric analysis (TGA) of the unmodified and modified isosorbide and sorbitol as well as the (finished) cotton fabrics were performed to examine the thermal decomposition and char formation. All samples were conditioned at 23 °C and 50% relative humidity prior to analysis. Measurements were performed in air and nitrogen ramping the temperature from 30 to 600 °C with a rate of 10 °K/min. The thermograms were analysed using Universal Analysis Software (v5.5.24) from TA Instruments (Asse, Belgium).

Cone calorimetry tests were performed with a device manufactured by Dr.-Ing. Georg Wazau Mess- + Prüfsysteme GmbH (Berlin, Germany). The tests were carried out by using an incident heat flux of 25 kW/m^2^. The sample size was 100 mm × 100 mm × thickness of the samples. The tested materials were wrapped with aluminium foil according to ISO 5660-1:2015. Because the samples were only around 0.5 mm thin, a monolith (calcium silicate) plate was placed underneath to ensure an even surface. Measured parameters included time of ignition (t_ign_), peak heat release rate (PHRR), total heat release (THR), total some release (TSR) and residual mass. All samples were measured threefold.

The burning behaviour of the cotton fabrics was assessed via ISO 15025 (surface and edge ignition). This standard is used to determine the flame spread properties of vertically oriented materials, when subjected to a small defined flame. A defined flame from a specified burner is applied for 10 s to the surface or the bottom edge of textile specimens which are vertically oriented. Criteria such as afterflame time (flaming after removal of ignition source), afterglow (persistence of glowing combustion of material), formation of molten or flaming debris, hole formation and flame on edge are assessed. Wash fastness was evaluated according to ISO 6330 (washing method 6A) at 40 °C. Wascator FOM 71 (type A) from Electrolux Professional (Stockholm, Sweden) was applied as apparatus and the used detergent was an ECE detergent (type 3). The samples were flat dried at room temperature. Flame retardancy was assessed after five washing cycles.

## 3. Results and Discussion

### 3.1. Structural Characterisation of Sorbitol, Isosorbide and the Modified Analogues

**Figure 1:**
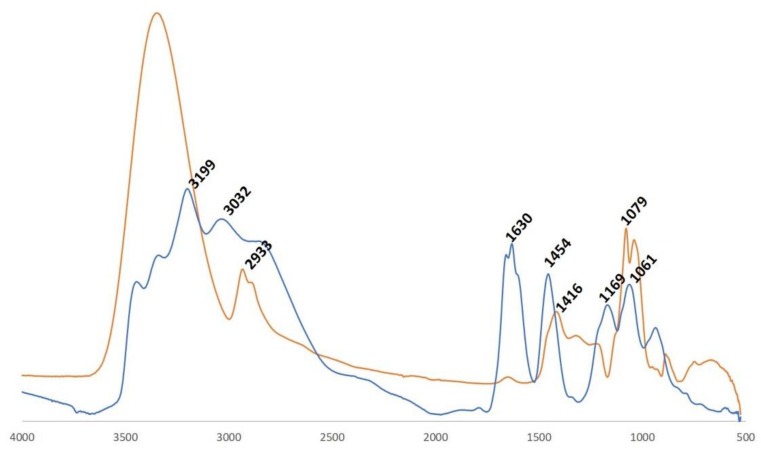
FT-IR spectra of sorbitol (orange) and modified sorbitol (blue).

The FT-IR spectra of sorbitol and modified sorbitol are presented in Figure 1. In the case of sorbitol, characteristic bands were observed at 1079 and 1416 cm^−1^. The band located at 1079 cm^−1^ is attributed to C–OH stretching vibrations, while the band at 1416 cm^−1^ is due to bending vibrations of OH bonds. The band at 2933 cm^−1^ is caused by CH stretching. A strong broad band around approximately 3400 cm^−1^, assigned to OH–vibrations, was observed in the FT-IR spectrum of sorbitol [28]. This band disappeared in the FT-IR spectrum of modified sorbitol, indicating that hydroxyl groups were converted. Further on, a few new characteristic bands appeared in the FT-IR spectrum of modified sorbitol. The band at 3199 cm^−1^ can be assigned to N–H stretching vibration in ammonium ion. The band at 1454 cm^−1^ can be attributed to deformation vibration of N–H in ammonium ion. The band at 1169 cm^−1^ is due to P=O stretching and the band at 1630 cm^−1^ can be assigned to bending HOH vibrations from absorbed water [29,30].

**Figure 2:**
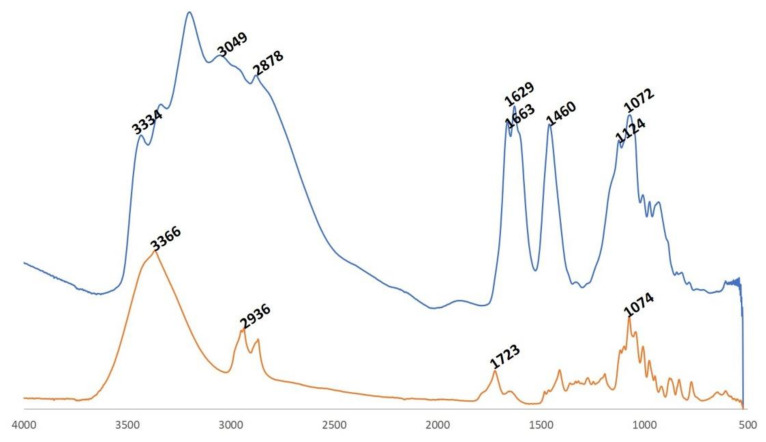
FT-IR spectra of isosorbide (orange) and modified isosorbide (blue).

The FT-IR spectra of isosorbide and modified isosorbide are presented in Figure 2. Isosorbide exhibited a characteristic strong band at 3366 cm^−1^ due to OH stretching vibration. The band at 2936 cm^−1^ is assigned to C–H stretching. The band located at 1074 cm^−1^ is attributed to C–OH stretching vibrations. New characteristics band appeared in the FT-IR spectrum of modified isosorbide of which the bands at 3201 and 3049 cm^−1^ can be assigned to N–H stretching in ammonium ion. The band at 1460 cm^−1^ can be attributed to deformation vibration of N–H in ammonium ion. The band at 1629 cm^−1^ is due to bending HOH vibrations from absorbed water.

**Figure 3:**
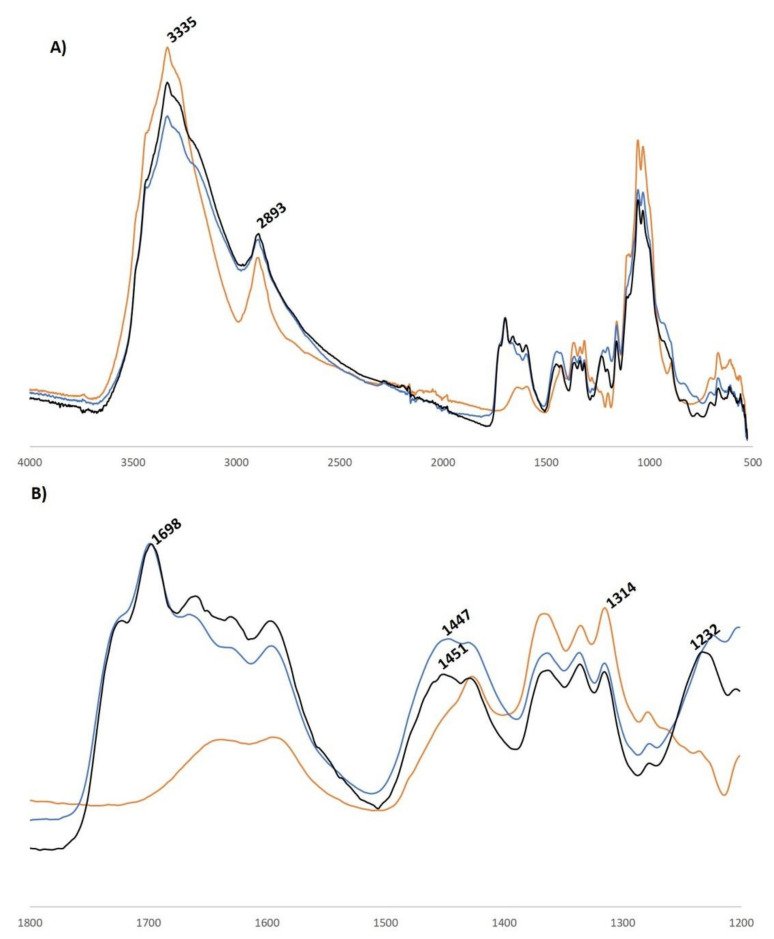
FT-IR spectra of cotton (orange), Co-P-isosorbide (black) and Co-P-sorbitol (blue) from 500–4000 cm^−1^ (**A**) and a detailed view from 1200–1800 cm^−1^ (**B**).

In Figure 3, the FT-IR spectrum of untreated cotton is presented along with the FTIR-spectra of cotton treated with 40% modified isosorbide (Co-P-isosorbide) or 40% modified sorbitol (Co-P-sorbitol). Untreated and treated cotton showed strong characteristic bands at 3335 and 2893 cm^−1^, assigned to OH stretching and C–H stretching vibrations, respectively. Treated cotton fabrics exhibited new characteristic bands. The band at 1698 cm^−1^ is allocated to C=O stretching due to oxidation of cotton. The band at 1232 cm^−1^ can be attributed to P=O stretching and the band at 1447 cm^−1^ is caused by deformation vibration of N–H in the ammonium ion.

The presence of phosphorus in the different materials was assessed via XRF analysis. No phosphorus was detected in case of sorbitol, isosorbide and untreated cotton. Modified sorbitol contained 12 wt% phosphor, while Co-P-sorbitol contained 4 wt% phosphor, respectively. Modified isosorbide contained 8.6 wt% phosphor, while Co-P-isosorbide contained 3.8 wt% phosphor, respectively. As expected, modified sorbitol showed higher phosphorus content, since sorbitol has more hydroxyl groups which could be converted in phosphate groups via reaction with phosphoric acid.

### 3.2. Thermal Degradation Behavior

**Figure 4:**
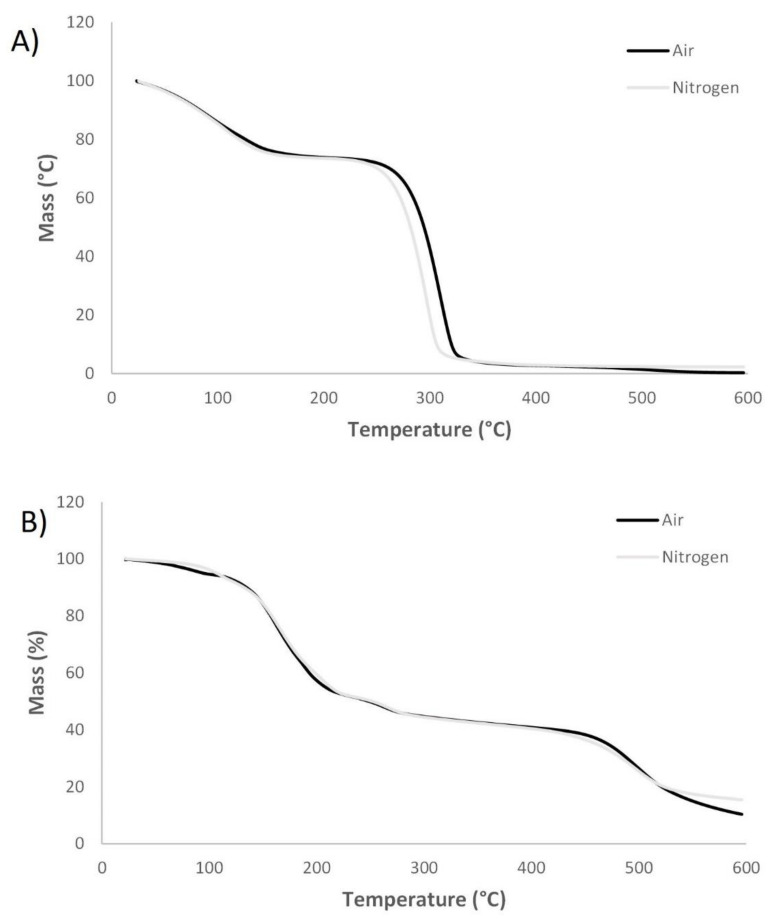
TGA curves from unmodified sorbitol in air and nitrogen (**A**) and modified sorbitol in air and nitrogen (**B**).

The thermal properties of sorbitol, isosorbide and the corresponding modified molecules were analysed via TGA in air and nitrogen atmosphere. Figure 4 demonstrates the TGA curves of sorbitol and modified sorbitol. Two mass losses were observed for sorbitol in both air and nitrogen atmosphere. The initial loss corresponds to water evaporation (25%). The second weight loss corresponds to evaporation of sorbitol (boiling point: 296 °C). The TGA curves for modified sorbitol in air and nitrogen atmosphere exhibited different mass losses, besides evaporation of water initially. According to Davies et al. mass losses could be attributed to degradation of the crystalline form to a more stable form and release of ammonia and phosphoric acids and cross-linking [31]. The weight residue of modified sorbitol in air atmosphere was 31%, indicating potential charring and flame retardancy.

**Figure 5:**
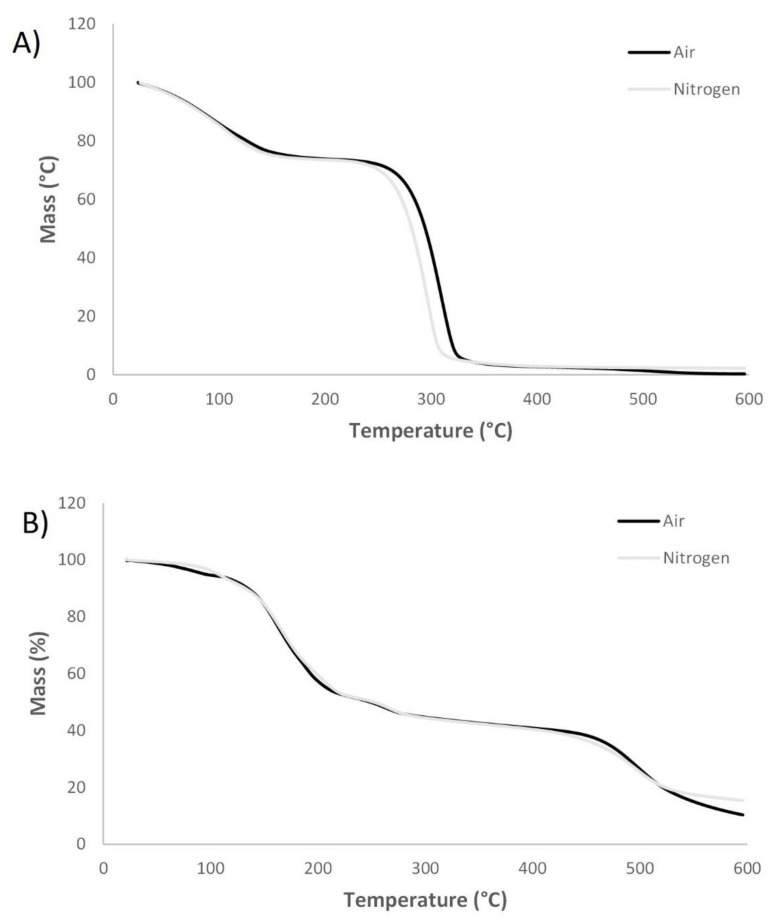
TGA curves of unmodified isosorbide in air and nitrogen (**A**) and modified isosorbide in air and nitrogen (**B**).

Figure 5 demonstrates the TGA curves of isosorbide and modified isosorbide in air and nitrogen. In both, air as well as nitrogen atmosphere, isosorbide started to degrade very rapidly at approximately 150 °C, resulting in no residue at 200 °C. The TGA curves of modified isosorbide in air and nitrogen atmosphere were similar. Initially, a weight loss was observed due to evaporation of water, followed by mass losses which might indicate degradation of the crystalline form and release of ammonia or phosphoric acid. The weight residue of modified isosorbide in air atmosphere was 27%, indicating potential charring and flame retardancy.

**Figure 6:**
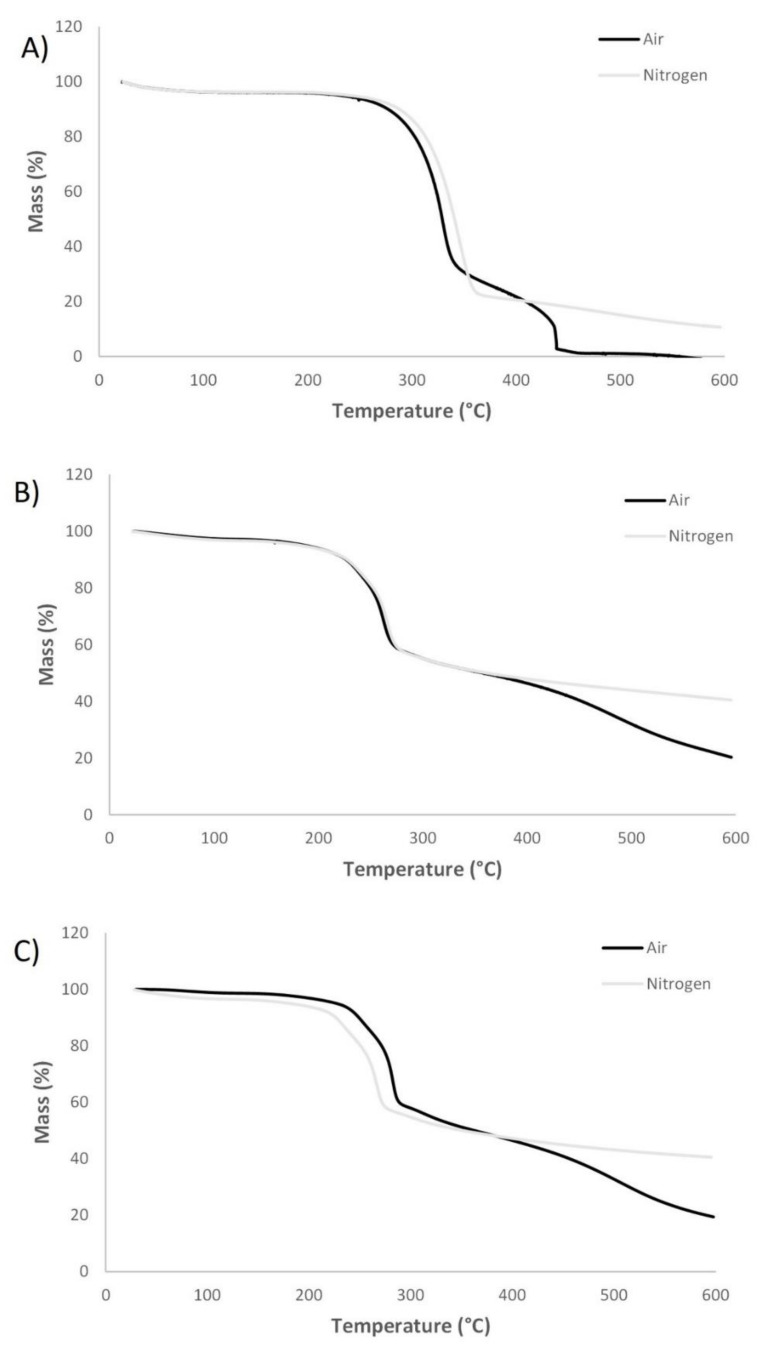
TGA curves of cotton in air and nitrogen (**A**), cotton finished with modified sorbitol in air and nitrogen (**B**) and cotton finished with modified isosorbide in air and nitrogen (**C**).

TGA analysis was also performed on Co-P-isosorbide and Co-P-sorbitol (Figure 6). In both air and nitrogen atmosphere cotton showed a minor mass loss initially due to water evaporation. The main degradation in nitrogen atmosphere occurred in the range of 270–360 °C, which is attributed to α-cellulose degradation (dehydration and decarboxylation reactions resulting in combustive gasses) [32,33]. A char residue of approximately 10% was obtained in nitrogen, while no char was present in air. In air, a second weight loss was observed, which can be assigned to oxidative degradation of char formed previously, resulting in a lower residue in air compared to nitrogen atmosphere [26]. Cotton finished with modified sorbitol showed analogous degradation to untreated cotton including a minor mass loss due to water evaporation. However, degradation began at lower temperature. In air, oxidation of char was observed leading to a lower char residue in air (20%) compared to nitrogen (40%). From thermal degradation analysis, it was clear that modified sorbitol increased the formation of char residue at 600 °C for the cotton fabric, indicating potential FR effect. Cotton finished with modified isosorbide exhibited similar TGA curves (in both, air and nitrogen atmosphere) than cotton finished with modified sorbitol including equal amounts of char residue at 600 °C. A lower char residue in air (19%) compared to nitrogen (40%) was observed due to oxidation of char. Increased char residue at 600 °C demonstrates the potential FR effect of cotton finished with modified isosorbide.

### 3.3. Cone Calorimetry

**Figure 7:**
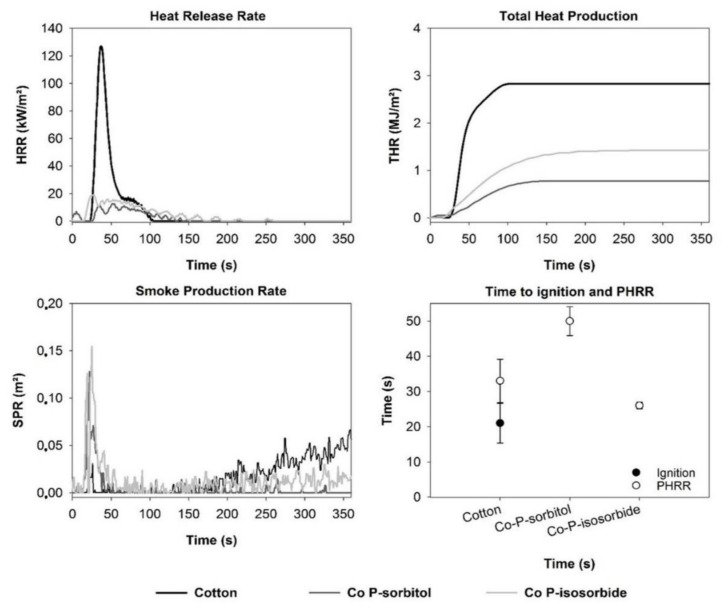
Cone calorimetry data of cotton (black), Co-P-sorbitol (dark grey) or Co-P-isosorbide (light grey).

**Figure 8:**
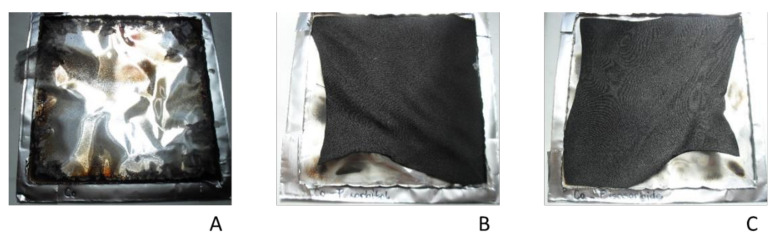
Comparison of untreated cotton (**A**) and Co-P-sorbitol (**B**) and Co-P-isosorbide (**C**) after cone calorimetry measurements.

Cone calorimetry measurements were performed on cotton treated with 40% modified sorbitol and 40% modified isosorbide using untreated cotton as reference material. Figure 7 summarizes the results of heat release rate (HRR), total heat release (THR), smoke production rate (TSP) and time to ignition and peak heat release rate (PHRR). For the untreated cotton fabric, a sharp peak which is typical for thin films was obtained for HRR [34]. The whole sample is pyrolysed at the same time. In this special case, the HRR becomes dependent on their total fire load. In contrast, it was not possible to ignite the biobased samples treated with the biobased FR under the chosen test parameters for energy and distance between the heater and the sample. Consequently, no time to ignition could be determined for Co-P-sorbitol and Co-P-isosorbide. Nevertheless, a heat release was detected. The released heat and the maximal released heat were markedly less when compared to the cotton reference. Pure cotton had a THR of 2.79 ± 0.09 MJ/m^2^ while Co-P-sorbitol and Co-P-isosorbide treated cotton showed THR of 0.62 ± 0.28 MJ/m^2^ and 1.22 ± 0.30 MJ/m^2^, respectively. Even if the samples were not ignited, they were affected by the exposed heat. Figure 8 demonstrates that charring of cotton treated with biobased FR took place instead of complete combustion as seen for cotton. These findings correspond to the results of TGA measurements where increasing char formation was found for cotton fabrics treated with modified biobased FR. These results support the fact that modified sorbitol and isosorbide have an influence on FR properties.

Furthermore, similar values for the smoke production rate were obtained for all three investigated samples (cotton, Co-P-sorbitol and Co-P-isosorbide). The charring of the FR containing samples lead to a release of smoke without burning (contrary to untreated cotton).

### 3.4. Burning Behavior

The flame retardant properties of cotton and finished cotton fabrics were evaluated according to ISO 15025 (surface and edge ignition). The results are shown in Table 1. Untreated cotton fabric didn’t show any flame retardancy and failed ISO 15025 test (flame on edge and flaming debris). After surface ignition, cotton fabric showed a very long afterglow time. Co-P-isosorbide and Co-P-sorbitol passed both surface and edge ISO 15025. Flame-retardant properties were excellent since no afterflame and afterglow were observed.

The durability of the flame retardant properties were assessed after five washing cycles at 40 °C (Table 2). Washed Co-P-isosorbide and Co-P-sorbitol showed limited afterflame and afterglow after surface ignition without the flame reaching the edge. In case of edge ignition, more distinct differences were noticed in the fire behaviour of the washed cotton samples. Co-P-sorbitol and Co-P-isosorbide showed minor afterflame and afterglow time and passed the edge ignition test since the flame self-extinguished before reaching the edge. However, Co-P-isosorbide samples showed longer afterflame and afterglow time compared to Co-P-sorbitol samples, probably due to lower phosphorus content.

## 4. Conclusions

Isosorbide and sorbitol based FR were synthesized and applied on cotton fabrics. The biobased FR were characterised via FT-IR, TGA and XRF. XRF analysis demonstrated the presence of phosphorus. Modified sorbitol contained more phosphorus compared to modified isosorbide (12% versus 8.6%) since sorbitol has more hydroxyl groups which could be converted in phosphate groups via reaction with phosphoric acid. FT-IR analysis revealed new characteristic bands in the FT-IR spectra of modified isosorbide and modified sorbitol, owing to the presence of phosphorus and nitrogen. The residual weight measured during TGA-analysis at 600 °C in air for both, modified sorbitol and modified isosorbide, was 31% and 27%, respectively. Cotton finished with modified sorbitol or modified isosorbide had a residual weight of approximately 20% in air, while untreated cotton had no residual weight indicating a potential FR effect (char forming effect) of the modified substances due to the presence of phosphate and ammonia groups in modified sorbitol and modified isosorbide. Co-P-sorbitol and Co-P-isosorbide passed ISO 15025 FR test initially and after five washing cycles at 40 °C, while the untreated cotton fabric failed to pass ISO 15025. By means of cone calorimeter measurements, no ignition of cotton treated with the modified FR was detected. The released heat and the maximal released heat were also less when compared to the cotton reference. Pure cotton had a THR of 2.79 ± 0.09 MJ/m^2^ while Co-P-sorbitol and Co-P-isosorbide treated cotton showed THR of 0.62 ± 0.28 MJ/m^2^ and 1.22 ± 0.30 MJ/m^2^, respectively. Nevertheless, the finished samples were affected by the heat supply. A charring of FR containing samples was observed, confirming the results of TGA measurements. At the end, biobased modified sorbitol/isosorbide cotton show good flame retardant properties and good durability against washing. This new improvement will be used in textile industry tending to more sustainable, environmentally friendly and biobased products.

## Figures and Tables

**Table 1:** Overview of ISO 15025 results.

Surface Ignition						
Sample	Afterflame (s)	Afterglow (s)	Flaming/Molten Debris	Flame on Edge	Hole on Edge	Hole Formed
Cotton	63	1073	yes	yes	yes	yes
Cotton + 40% modified isosorbide	0	0	no	no	no	no
Cotton + 40% modified sorbitol	0	0	no	no	no	no
**Edge ignition**						
**Sample**	**Afterflame (s)**	**Afterglow (s)**	**Flaming/molten debris**	**Flame on edge**		
Cotton + 30% modified isosorbide	0	0	no	no		
Cotton + 40% modified isosorbide	0	0	no	no		
Cotton + 40% modified sorbitol	0	0	no	no		

**Table 2:** Overview of ISO 15025 results after five washing cycles (40 °C).

Surface Ignition						
Sample	Afterflame (s)	Afterglow (s)	Flaming/Molten Debris	Flame on Edge	Hole on Edge	Hole Formed
Cotton	145	23	no	yes	yes	yes
Cotton + 40% modified isosorbide	15	12	no	no	no	no
Cotton + 40% modified sorbitol	11	18	no	no	no	no
**Edge ignition**						
**Sample**	**Afterflame (s)**	**Afterglow (s)**	**Flaming/molten debris**	**Flame on edge**		
Cotton	27	21	no	yes		
Cotton + 40% modified isosorbide	13	11	no	no		
Cotton + 40% modified sorbitol	4	18	no	no		

## Data Availability

The data presented in this study are available on request from the corresponding author.
